# Guidelines for the use and interpretation of Alzheimer’s disease biomarkers in clinical practice in Brazil: recommendations from the Scientific Department of Cognitive Neurology and Aging of the Brazilian Academy of Neurology

**DOI:** 10.1590/1980-5764-DN-2024-C001

**Published:** 2024-11-11

**Authors:** Adalberto Studart-Neto, Breno José Alencar Pires Barbosa, Artur Martins Coutinho, Leonardo Cruz de Souza, Lucas Porcello Schilling, Mari Nilva Maia da Silva, Raphael Machado Castilhos, Paulo Henrique Ferreira Bertolucci, Wyllians Vendramini Borelli, Hélio Rodrigues Gomes, Gustavo Bruniera Peres Fernandes, Maira Tonidandel Barbosa, Marcio Luiz Figueredo Balthazar, Norberto Anízio Ferreira Frota, Orestes Vicente Forlenza, Jerusa Smid, Sonia Maria Dozzi Brucki, Paulo Caramelli, Ricardo Nitrini, Eliasz Engelhardt, Elisa de Paula França Resende

**Affiliations:** 1 Academia Brasileira de Neurologia, Departamento Científico de Neurologia Cognitiva e do Envelhecimento, São Paulo SP, Brazil.; 2 Universidade de São Paulo, Faculdade de Medicina, Hospital das Clínicas, Departamento de Neurologia, Grupo de Neurologia Cognitiva e do Comportamento, São Paulo SP, Brazil.; 3 Universidade Federal de Pernambuco, Hospital das Clínicas, Recife, Centro de Ciências Médicas, Recife PE, Brazil.; 4 Universidade Federal de Pernambuco, Empresa Brasileira de Serviços Hospitalares, Hospital das Clínicas, Departamento de Neurologia, Recife PE, Brazil.; 5 Universidade de São Paulo, Faculdade de Medicina, Hospital das Clínicas, Instituto de Radiologia, Centro de Medicina Nuclear, Laboratório de Investigação Médica (LIM 43), São Paulo SP, Brazil.; 6 Hospital Sírio-Libanês, Medicina Nuclear e Serviço de PET-CT, São Paulo SP, Brazil.; 7 Universidade Federal de Minas Gerais, Faculdade de Medicina, Unidade de Neurologia Cognitiva e do Comportamento, Belo Horizonte MG, Brazil.; 8 Pontifícia Universidade do Rio Grande do Sul, Escola de Medicina, Serviço de Neurologia, Porto Alegre RS, Brazil.; 9 Hospital Nina Rodrigues, Serviço de Neuropsiquiatria, São Luís MA, Brazil.; 10 Hospital de Clínicas de Porto Alegre, Serviço de Neurologia, Centro de Neurologia Cognitiva e Comportamental, Porto Alegre RS, Brazil.; 11 Universidade Federal de São Paulo, Escola Paulista de Medicina, Departamento de Neurologia e Neurocirurgia, São Paulo SP, Brazil.; 12 Universidade Federal do Rio Grande do Sul, Instituto de Ciências Básicas da Saúde, Departamento de Ciências Morfológicas, Porto Alegre RS, Brazil.; 13 Universidade de São Paulo, Faculdade de Medicina, Hospital das Clínicas, Laboratório de Líquido Cefalorraquidiano, São Paulo SP, Brazil.; 14 Universidade de São Paulo, Faculdade de Medicina, Laboratório de Investigação Médica (LIM 15), São Paulo SP, Brazil.; 15 Departamento Científico de Líquido Cefalorraquiano, Academia Brasileira de Neurologia, São Paulo SP, Brazil.; 16 Hospital Israelita Albert Einstein, Laboratório Clínico, São Paulo SP, Brazil; 17 Universidade Estadual de Campinas, Faculdade de Ciências Médicas, Departamento de Neurologia, Campinas SP, Brazil.; 18 Hospital Geral de Fortaleza, Serviço de Neurologia, Fortaleza CE, Brazil.; 19 Universidade de Fortaleza, Fortaleza, CE, Brazil.; 20 Universidade de São Paulo, Faculdade de Medicina, Hospital das Clínicas, Instituto de Psiquiatria, Laboratório de Neurociências, São Paulo SP, Brazil.; 21 Universidade Federal do Rio de Janeiro, Instituto de Neurologia Deolindo Couto, Rio de Janeiro RJ, Brazil.; 22 Universidade Federal do Rio de Janeiro, Instituto de Psiquiatria, Rio de Janeiro RJ, Brazil.

**Keywords:** Alzheimer Disease, Cognitive Dysfunction, Diagnosis, Biomarkers, Neuroimaging, Doença de Alzheimer, Disfunção Cognitiva, Diagnóstico, Biomarcadores, Neuroimagem

## Abstract

In recent years, the diagnostic accuracy of Alzheimer’s disease has been enhanced by the development of different types of biomarkers that indicate the presence of neuropathological processes. In addition to improving patient selection for clinical trials, biomarkers can assess the effects of new treatments on pathological processes. However, there is concern about the indiscriminate and poorly supported use of biomarkers, especially in asymptomatic individuals or those with subjective cognitive decline. Difficulties interpreting these tests, high costs, and unequal access make this scenario even more challenging in healthcare. This article presents the recommendations from the Scientific Department of Cognitive Neurology and Aging of the Brazilian Academy of Neurology (*Departamento Científico de Neurologia Cognitiva e Envelhecimento da Academia Brasileira de Neurologia*) regarding the rational use and interpretation of Alzheimer’s disease biomarkers in clinical practice. The clinical diagnosis of cognitive-behavioral syndrome is recommended as the initial step to guide the request for biomarkers.

## RESUMO

Nos últimos anos, a precisão diagnóstica da doença de Alzheimer tem sido aprimorada pelo desenvolvimento de diferentes tipos de biomarcadores que indicam a presença dos processos neuropatológicos. Além de melhorar a seleção de pacientes para ensaios clínicos, os biomarcadores podem avaliar os efeitos de novos tratamentos nos processos patológicos. No entanto, há preocupação com o uso indiscriminado e mal fundamentado de biomarcadores, especialmente em indivíduos assintomáticos ou com declínio cognitivo subjetivo. As dificuldades na interpretação desses testes, os altos custos e o acesso desigual tornam esse cenário ainda mais desafiador no cuidado assistencial. Neste artigo, são apresentadas as recomendações do Departamento Científico de Neurologia Cognitiva e Envelhecimento da Academia Brasileira de Neurologia quanto ao uso racional e interpretação de biomarcadores da doença de Alzheimer na prática clínica. O diagnóstico clínico da síndrome cognitivo-comportamental é recomendado como o passo inicial para orientar a solicitação de biomarcadores.

## INTRODUCTION

In recent years, the diagnostic accuracy of Alzheimer’s disease (AD) has been enhanced by the development of different types of biological markers (biomarkers) that indicate the presence of the neuropathological processes associated with AD. Beyond being very important for the research of new therapies, by improving the selection of patients for clinical trials, biomarkers are able to access the effects of new treatments on pathological processes much earlier than neuropsychological and functional evaluations^1^.

Since 2011, the use of biomarkers as a tool to increase the diagnostic accuracy of the symptomatic phases of AD in clinical practice has been debated. Subsequently, biomarkers began to be used to optimize the selection of patients and asymptomatic individuals (pre-clinical phase) for clinical trials and research. In 2018, biomarkers were definitively incorporated into research (biological diagnosis). In recent years, much progress has been achieved in molecular neuroimaging methods^2^ and in the techniques for measuring biomolecules in body fluids — cerebrospinal fluid (CSF) and plasma^3^. More recently, in 2024, a workgroup from the Alzheimer’s Association (AA) updated the 2011 and 2018 diagnostic criteria, reaffirming that the diagnosis of AD should be biological, based on biomarkers, besides having recommended biomarkers for biological staging of the disease^4^.

However, there is concern about the indiscriminate and poorly founded use of biomarkers, especially in asymptomatic individuals or those with subjective cognitive decline (SCD). The development of plasma biomarkers and greater access to these tests make it even more necessary to exercise caution in requesting these biomarkers, as they will become increasingly available. Besides the intrinsic difficulties in interpreting these tests, this scenario is also challenging due to their high costs and unequal access in Brazil^5^ and even in high-income countries^6^. Concerned about the inappropriate use of biomarkers, a European consensus established criteria for their application in clinical practice for mild cognitive impairment (MCI) and mild dementia^7^.

Given the recent advances in the field of AD biomarkers, the main objective of this article was to establish recommendations from the Scientific Department of Cognitive Neurology and Aging of the Brazilian Academy of Neurology (*Departamento Científico de Neurologia Cognitiva e Envelhecimento da Academia Brasileira de Neurologia*) regarding the rational use and interpretation of AD biomarkers in clinical practice. These recommendations may serve as a guide for physicians who assist patients with cognitive impairment and dementia. Therefore, the diagnosis of the patient’s cognitive-behavioral syndrome through anamnesis and cognitive, behavioral, and functional clinical evaluations should always be the initial step to guide the request for biomarkers.

## METHODS

The recommendations were prepared by a group of medical specialists and researchers in AD and other dementias (neurologists, geriatricians, psychiatrists, nuclear medicine physicians, and clinical pathologists). A non-systematic review of the literature was conducted from searches in the MEDLINE, Scopus, SciELO, and LILACS databases until July 2024, using the descriptors “Alzheimer’s disease” or “biomarkers”. We selected mainly articles published in the last ten years as well as relevant older publications. The authors then met several times for discussion, and consensus points were included in the recommendations.

## DEFINITION OF BIOMARKERS

Biomarkers in medicine can be defined as measures or indicators of normal or pathological physiological processes or responses to a therapeutic intervention^8^. The ideal biomarker for AD should reflect the neuropathological characteristics of the disease; demonstrate diagnostic accuracy in pathologically confirmed cases; have a sensitivity of at least 85%; have specificity to differentiate AD from cognitively healthy individuals and other diseases causing cognitive impairment of at least 75%; indicate the real presence of AD and not merely an increased risk; allow monitoring of disease severity or progression; indicate the effectiveness of the therapeutic intervention; be non-invasive; and be economically accessible^9,10^.

## GENESIS OF BIOMARKERS - WHERE DO THEY COME FROM?

The neuropathological signature of AD includes amyloid plaques formed by extracellular deposits of amyloid beta (Aβ) peptides and neurofibrillary tangles (NFTs) formed by intracellular aggregates of hyperphosphorylated tau proteins (p-tau), inflammatory reaction, astrocyte and microglial activation, as well as synaptic and neuronal loss^11,12,13,14^.

### The amyloid cascade hypothesis

Amyloid plaques, composed of amyloid fibrils in a beta-sheet conformation, are considered the primary cause of AD according to the “amyloid cascade hypothesis”^15,16,17^. This theory suggests that altered metabolism of the amyloid precursor protein (APP) by secretases (α, β, γ) leads to the release and aggregation of Aβ peptides (mainly Aβ_40_ and Aβ_42_), causing microglial activation, astrocytic reactivity, neuroinflammation, oxidative stress, p-tau hyperphosphorylation, and NFTs^18,19,20^. The hypothesis explains the genetic forms of AD, but not the far more common sporadic forms.

### The amyloid beta peptide and amyloid plaques

Aβ peptides initially appear as monomers that can form various aggregates, from small oligomers to larger protofibrils and fibrils. While amyloid fibrils are insoluble and form amyloid plaques characteristic of AD, oligomers are soluble and can spread throughout the brain. Under physiological conditions, most Aβ peptides are Aβ_40_, and less than 10% are Aβ_42_, which is more prone to aggregation and is associated with the formation of amyloid oligomers, fibrils, and plaques^21^. Aβ production is balanced by degradation, clearance, transport, and deposition processes. Around 10–15% of Aβ may enter the CSF and bloodstream from the brain^22^.

Amyloid fibrils aggregate with other molecules, forming plaques in the brain’s extracellular space, predominantly near synapses, in three types: diffuse, dense-core, and neuritic plaques^11,13,14,23^. Plaques gradually grow in the brain’s interstitial space from continuous extracellular deposition of Aβ peptides at “seed” sites.

### Neurofibrillary pathology and phosphorylated tau protein

Neurofibrillary pathology in AD includes NFTs, neuropil threads, and dystrophic neurites^14,24,25^. NFTs are abnormal bundles of p-tau in neurons^24^.

There are six tau isoforms in the adult human brain^26,27^, with 3R and 4R isoforms present in approximately equal levels^28,29^. In AD, NFTs contain both 3R and 4R isoforms. Other human tauopathies present pathological aggregates of either 3R or 4R tau isoforms^27,30,31^. P-tau, primarily found in neurons, stabilizes microtubules ^27,31^ and is regulated by phosphorylation. Pathological phosphorylation of tau reduces its microtubule affinity, leading to cytoskeletal destabilization^32,33^.

P-tau can be released into interstitial fluid, CSF, and blood, where it can be detected as indirect evidence of AD^32,34^. Pathological p-tau can be phosphorylated at several specific amino acids, such as 181, 217, and 231.

## CLINICAL AND BIOLOGICAL DIAGNOSES OF ALZHEIMER’S DISEASE: THE EVOLUTION OF DIAGNOSTIC CRITERIA

Although the description of the disease that bears his name was published in 1907 by the German neuropsychiatrist and neuropathologist Dr. Alois Alzheimer^35^, the first diagnostic criteria were proposed only in 1984 by McKhann et al.^36^. At that time, they considered only the amnestic presentation of AD and admitted diagnosis only in the dementia phase. In 2011, new criteria for diagnosing AD were published by the National Institute on Aging and the Alzheimer’s Association (NIA-AA)^37,38,39^. In contrast to previous recommendations, the 2011 NIA-AA criteria began to admit prodromal (i.e., pre-dementia) phases and the possibility of non-amnestic variants of AD ^37,39^. The 2011 criteria anticipated the use of biomarkers for the etiological diagnosis of dementia syndrome^37^ or MCI^38^, proposing the terms “dementia due to AD”^37^ and “MCI due to AD”^38^. Subsequent studies showed that these 2011 clinical criteria, without biomarkers, had a sensitivity of 70.9–87.3% and a low specificity of 44.3–70.8% when compared to pathological diagnosis^40^. In another study, the use of biomarkers led to a change in diagnosis in 36% of dementia cases with uncertain diagnosis^41^. It is estimated that in specialized memory centers, diagnostic errors range from 25–30% when biomarkers are not used^42^.

Subsequently, in 2018, the NIA-AA proposed a new conceptual model for the biological definition of AD based on biomarkers according to the Amyloid-Tau-Neurodegeneration - AT(N) system. In this 2018 model, the presence or absence of symptoms would not be necessary for AD diagnosis, with biomarkers A and T being sufficient. According to this model, AD is biologically defined by evidence of disease-related pathology through biomarkers, independent of the clinical syndrome. The goal was to improve AD diagnosis specificity and apply it in clinical research, while the 2011 criteria would remain valid for use in clinical care^43,44^.

According to this proposal, AD is defined by the positivity of biomarkers for amyloid (A+) and p-tau (T+), regardless of the presence or absence of neurodegeneration (N+ or N-). Practically, the biological diagnosis of AD would require low concentrations of Aβ_42_ and high concentrations of p-tau in the CSF or positivity on amyloid-PET (positron emission tomography) and tau-PET (Table 1). When there is only the presence of Aβ_42_ (A+) without evidence of tau pathology (T-), the term used is “Alzheimer’s pathologic change,” representing the initial stage of the AD pathological spectrum according to the amyloid cascade hypothesis. Conversely, when there is positivity for p-tau (T+) and/or neurodegeneration (N+), but negativity for amyloid pathology (A), the diagnosis of Alzheimer’s pathology should be excluded, and it would be termed “suspected non-Alzheimer’s pathophysiology” (SNAP).

**Table 1:** Diagnostic categories defined by the Amyloid-Tau-Neurodegeneration system within the *continuum* of the biological definition of Alzheimer’s disease, according to the 2018 National Institute on Aging and the Alzheimer’s Association Research Framework^43,44^.

ATN	Category according to biomarkers profile
A-T-N-	Normal Alzheimer’s disease biomarkers
A+T-N-	Alzheimer’s disease pathological changes
A+T+N-	Alzheimer’s disease (without neurodegeneration)
A+T+N+	Alzheimer’s disease (with neurodegeneration)
A+T-N+	Alzheimer’s disease pathological changes + non-Alzheimer pathology
A-T+N-	Suspected non-Alzheimer’s pathophysiology (SNAP)
A-T-N+	Suspected non-Alzheimer’s pathophysiology (SNAP)
A-T+N+	Suspected non-Alzheimer’s pathophysiology (SNAP)

Abbreviations: ATN, Amyloid-Tau-Neurodegeneration.

Although the 2018 criteria consider only biomarkers for AD diagnosis and not for the clinical syndrome, the model recognizes that AD can manifest along a *continuum* from an asymptomatic phase, through stages of SCD and MCI, to the dementia phase. In 2018, the authors of this diagnostic consensus made it clear that this biological definition, independent of the presence of cognitive symptoms, should only be used in research and not in clinical practice due to the lack of effective treatments for preclinical diagnoses of the disease.

In 2024, an AA workgroup published an update to the 2018 criteria; before being published, an initial draft of the article was made public for scientific contributions for a few months^4^. The motivations for updating the 2018 criteria were:

- Approval of anti-amyloid drugs by regulatory agencies, which can only be used in patients with a confirmed AD diagnosis by biomarkers and in the early stages of the disease;
- Recent advances in the development of plasma biomarkers; and
- Evidence that fluid and molecular neuroimaging biomarkers are not equivalent and constitute different categories.

The goal of the new criteria is to establish AD diagnosis by incorporating new advances in biomarkers, reinforcing the idea that the definition of AD is biological and that symptoms are not necessary for its diagnosis. Another proposed update is that biomarkers be used not only for diagnosis but also for staging and monitoring treatment response^4^. Although the authors state that these new criteria can inform diagnosis in both research and clinical care, they caution that these criteria are not intended to be step-by-step guidelines for clinical practice or treatment protocols. The authors also recognize that these new criteria may not be operationalizable in many medical centers, even in high-income countries^4^.

The 2024 AA workgroup criteria suggest dividing biomarkers into three categories (Table 2):

- Core (or specific) biomarkers of AD (biomarkers of amyloid and tau proteinopathies);
- Non-specific biomarkers involved in the pathogenesis of AD (biomarkers of neurodegeneration and inflammation); and
- Biomarkers of common co-pathologies associated with AD (e.g., alpha-synuclein and cerebrovascular disease)^4^.

**Table 2:** New categorization of Alzheimer’s disease fluid and molecular neuroimaging biomarkers according to the 2024 Alzheimer’s Association Workgroup criteria^4^.

Core AD biomarkers (or specific AD biomarkers)	
Core 1	
A	Aβ proteinopathy: Aβ_42_ (CSF or plasma) and amyloid PET
T_1_	Soluble (or secreted) phosphorylated tau proteinopathy: p-tau 181, p-tau 217, p-tau 231(CSF or plasma)
Core 2	
T_2_	Insoluble (or aggregated) phosphorylated tau proteinopathy: p-tau205, MTBR-243 (CSF or plasma), and tau PET
Non-specific biomarkers involved in the pathogenesis of AD	
N	Neurodegeneration: NfL (CSF or plasma), anatomic MRI, and FDG PET
I	Astrocytic inflammation and reactivity: GFAP (CSF or plasma)
Common AD associated co-pathologies biomarkers	
V	Vascular (cerebrovascular disease): infarction or WMH on MRI (or CT)
S	Alfa-synuclein proteinopathy: αSyn-SAA (CSF)

Abbreviations: AD, Alzheimer’s disease; Aβ, amyloid beta; αSyn-SAA, alpha-synuclein seed amplification assay; CSF, cerebrospinal fluid; CT, computed tomography; FDG, fluorodeoxyglucose; GFAP, glial fibrillary acidic protein; MRI, magnetic resonance imaging; MTBR, microtubule-binding region; NfL, neurofilament light chain; PET, positron emission tomography; p-tau, ; WMH, white matter hyperintensity.

Tau biomarkers are divided into two categories: T_1_ and T_2_. This division is being proposed due to the chronological difference in the alterations of tau biomarkers^4^. Some p-tau isoforms (181, 217, 231) change earlier, around the same time that amyloid PET becomes positive^45,46,47^. Although these soluble p-tau analytes in CSF and plasma are tau biomarkers, they may indicate that the patient already has moderate/frequent amyloid plaques (high CERAD score, Consortium to Establish a Registry for Alzheimer’s Disease) and most of these cases are also in Braak stages III to VI, meeting the criteria for intermediate/high AD neuropathological change^4^.

On the other hand, tau PET and other tau analytes, such as p-tau205 and MTBR-tau243 (microtubule-binding region of tau-containing residue 243) change later, when fibrillar tauopathy is at Braak stages IV to VI. Hence, these biomarkers are termed T_2_, indicating insoluble (or aggregated) phosphorylated tau proteinopathy^46,47 48^. Thus, amyloid and T_1_ biomarkers are considered core 1 biomarkers, while T_2_ biomarkers are core 2. Core 1 biomarkers change in the early stages of AD (still in an asymptomatic phase), while core 2 biomarkers become abnormal later, when the first symptoms begin.

The 2024 AA workgroup criteria propose that an abnormality in one of the specific core 1 biomarkers (or a combination of them) is sufficient for the diagnosis of AD: amyloid PET, CSF Aβ_42/40_, CSF p-tau 181/Aβ_42_, or CSF t-tau/Aβ_42_ (Table 2). The workgroup raises the possibility that plasma biomarkers could be used for diagnosis in the future, as long as they have an accuracy equivalent to already approved CSF biomarkers or amyloid PET. Core 2 biomarkers alone are not sufficient for diagnosis, but the combination of core 1 and core 2 biomarkers can be used for AD biological staging^4^. The AA workgroup currently recommends against diagnostic testing in asymptomatic individuals outside the context of research studies.

These criteria were criticized by the scientific community, particularly by the European group, the International Working Group (IWG), which proposed a parallel diagnostic criterion^49^. The IWG does not consider the presence of biomarkers as a sole diagnosis of AD because many people have positive biomarkers but never develop clinical symptoms^49^. Additionally, the IWG argues that the presence of biomarkers is a risk factor for AD, but alone, does not allow for a diagnosis of the disease, in contrast to the criteria suggested by the AA in 2024. These differences reflect some of the criticisms about the AA’s proposed criteria, including the lack of access to biomarkers in resource-limited settings, leading to disparities in diagnostic access, uncertainty regarding the prognosis of “asymptomatic” disease, and difficulties in managing the AD nomenclature. Still, there is a significant stigma associated with the name “Alzheimer’s disease” due to its invariably progressive and irreversible nature once symptoms begin. Moreover, the impact of such a diagnosis on asymptomatic individuals or those in the early stages of the disease is unknown. Finally, despite the existence of anti-amyloid therapies that show some clinical effect, these drugs have modest efficacy, potentially serious and fatal adverse events, and high costs, which currently do not justify screening for AD in the population, as is done with other diseases like diabetes mellitus, colon or cervical cancer, that can be diagnosed in asymptomatic individuals.

## BIOMARKERS IN CEREBROSPINAL FLUID

The most widely validated and studied CSF biomarkers are the proteins that characterize AD: the Aβ_42_ peptide and p-tau. In CSF, AD is characterized by a reduction in Aβ_42_ and an increase in p-tau. This pattern is known as the AD pathology signature in CSF^50^.

Recently, Aβ_40_ has been included in the diagnostic biomarker panel for AD, as evidence shows that the Aβ_42_/Aβ_40_ ratio outperforms Aβ_42_ dosage alone, demonstrating better concordance with amyloid PET positivity. The Aβ_40_ peptide has high concentration levels in CSF and does not vary in AD, unlike Aβ_42_. This causes the Aβ_42_/Aβ_40_ ratio in CSF to gradually decrease as the disease progresses^51^.

A meta-analysis of 131 studies revealed that CSF concentrations of Aβ_42_ in AD patients decrease by about 56% from normal levels^52^. A steeper decline in CSF Aβ_42_ levels occurs at the onset of the disease’s pathological process, followed by relative stability as the disease evolves. In contrast, p-tau levels change slowly over the course of the disease^53^.

Most studies showed approximately 90% concordance in comparing amyloid PET and CSF Aβ_42_ levels in LCR^51^. In a longitudinal study with older individuals without dementia, Palmqvist et al. observed that those with low CSF Aβ_42_ levels and negative amyloid PET had a higher rate of PET positivity over time than individuals with normal Aβ_42_ levels^54^. This finding suggests that CSF changes precede amyloid PET changes, explaining why the concordance between the two biomarkers is below 100%. It is important to note that these studies involved asymptomatic individuals. In patients at the dementia stage, a higher concordance between amyloid PET results and CSF Aβ_42_ levels is observed.

The accumulation of p-tau is the other component that defines AD besides Aβ peptide. The main assays used to measure p-tau quantify p-tau181, which presented the best accuracy in differentiating AD from cognitively healthy controls or other degenerative dementias. Assays using p-tau231 and p-tau199 also demonstrated good accuracy^51^. The ratio of p-tau/Aβ_42_ levels has also proven useful in AD diagnosis, with a sensitivity of 91.6% and a specificity of 85.7%^55^.

More recently, several studies showed that p-tau217 is the phosphorylated tau isoform with the earliest changes^45,46,47^. Evidence indicated that p-tau217 concentrations in CSF and plasma increase at the same time amyloid PET becomes positive and before tau PET changes^45,46,47,56^. Compared to p-tau181, p-tau217 exhibited a better correlation with amyloid PET and better differentiated A+ individuals from A- individuals^57^. Additionally, p-tau217 proved to be a better marker of cognitive progression to dementia in patients with MCI than p-tau181^58^. On the other hand, p-tau205 changed later and was better associated with tau PET than with amyloid PET^57^.

Although the p-tau dosage in CSF serves as a biomarker for tau pathology, the soluble tau isoforms do not correlate with the load of insoluble p-tau aggregates (represented by NFTs). Recently, the measurement of MTBR-tau243 residues was investigated as a new CSF biomarker specific for insoluble tau aggregates^59^. Changes in MTBR-tau243 in CSF occur simultaneously with changes in tau PET^59^.

Total p-tau (t-tau) measures all isoforms of p-tau, regardless of phosphorylation state; therefore, is not specific to AD. In fact, elevated t-tau levels can be observed in other conditions involving neuronal damage, both degenerative (e.g., Creutzfeldt-Jakob disease) and acute non-degenerative (e.g., cerebrovascular accident). Thus, t-tau is considered a nonspecific biomarker of neurodegeneration. Recently, the measurement of t-tau in CSF has been increasingly replaced by other neurodegeneration markers, especially neurofilament light chain (NfL). Similar to t-tau, increased NfL concentrations are not specific to AD and can rise in other neurodegenerative disorders, such as amyotrophic lateral sclerosis (ALS) and frontotemporal dementia (FTD). NfL is promising as it may predict disease progression and potentially response to disease-modifying treatments^60^.

Recent CSF AD biomarkers time course study involving Chinese participants during the 20 years preceding clinical diagnosis of sporadic Alzheimer’s disease, showed changes in CSF biomarkers concentration, as follows: Aβ_42_, 18 years; Aβ_42_/Aβ_40_ ratio, 14 years; p-tau 181, 11 years; t-tau, 10 years; and NfL, 9 years. As cognitive impairment progressed, the changes in CSF biomarker levels in the AD group initially accelerated and then slowed^61^.

Despite numerous studies demonstrating the good accuracy of CSF biomarkers for AD, one of the major challenges has been the significant variability between laboratories^51,62^. This variability often leads clinicians to disregard CSF results when they conflict with the diagnostic hypothesis and to seek molecular neuroimaging methods as biomarkers. This variability primarily stems from errors and failures in the pre-analytical (sample collection, storage, and transportation) and analytical (actual execution of the test) phases. It is estimated that pre-analytical problems account for up to 70% of errors in clinical laboratories^63^. To address this issue, the AA, in partnership with the Neurochemistry Laboratory at the University of Gothenburg (Sweden), established an external quality control (QC) program in 2009. The program aims to monitor variations in biomarker tests across different locations and batches and to help the participating laboratories synchronize their procedures. This external QC program is free for participating laboratories, whether they are devoted to research or clinical activities^62^.

Among the core biomarkers for AD, the measurement of Aβ_42_ is the most affected by the pre-analytical phase. Falsely reduced results, or potential false positives, can occur due to the adsorption of the peptide onto the walls of the tubes used for CSF collection. The Aβ_42_/Aβ_40_ ratio shows less variation than the concentration of Aβ_42_ alone^62^. The recommendation is to use low-binding polypropylene tubes for the collection and storage of CSF, avoiding glass tubes. Other interfering factors include sample handling, tube transfers (aliquoting), freeze-thaw cycles, and storage temperature.

Recently, a working group led by the AA, consisting of experts from academia and industry, proposed a new simplified and standardized pre-analytical protocol for CSF collection, handling, and analysis for clinical routine use. This proposal came up after a comprehensive analysis of the impact of pre-analytical factors, including the type of tube, sample handling procedures, and storage/transport conditions^62^.

The most used methods for measuring biomarkers are manual immunoassays, specifically enzyme-linked immunosorbent assay (ELISA). Even when following the recommendations suggested by the AA, the coefficient of variation (CV) for this technique between laboratories can reach 15–25% (medium to high). Recently, single molecule array (SIMOA™) technology, which involves encapsulating individual molecules in femtoliter reaction chambers using paramagnetic markers, has also been used for biomarker measurement due to its ability to detect very small concentrations of substances in CSF and plasma.

Fully automated platforms have been developed in recent years. Bittner et al. published a 100% automated method based on electrochemiluminescence immunoassay^64^. Automated assays offer significant advantages over the ELISA methodology as they eliminate manual steps, reducing the possibility of human interference in the analytical process, and they exhibit superior performance (low CVs: Aβ_42_ ≤ 6%; p-tau and t-tau: ≤ 2.5%) with a shorter test execution time (~20 minutes). A recent study demonstrated that the ratios of different automated platforms (p-tau/Aβ_42_ and Aβ_42_/Aβ_40_) in CSF show excellent concordance (positive and negative) with amyloid PET classification^65^. These data suggest that the p-tau/Aβ_42_ and Aβ_42_/Aβ_40_ ratios provide similar clinical information in evaluating amyloid pathology, thereby bringing greater reliability to clinical practice results. However, these automated platforms are still costly. Two automated electrochemiluminescence platforms have received regulatory approval in the United States (through FDA, Food and Drug Administration) and in the European Union (EMA, European Medicines Agency) to date^66,67^. The approval of these two platforms was conditioned on achieving accuracy close to 90% compared to amyloid PET. The sensitivity of these platforms ranges from 88–97%, while specificity ranges from 84–89%^66,67^.

We strongly recommend that clinicians observe the methodology used in these measures and, whenever possible, choose laboratories that use fully automated platforms to minimize analytic bias.

Finally, it is important to emphasize that CSF analysis is recommended in cases of rapidly progressive dementia, early-onset dementia, or atypical dementias, to exclude infectious, inflammatory autoimmune, or neoplastic diseases^38^. In these cases, the CSF examination must include a red blood cell and nucleated cell count, differential leukocyte analysis, total protein measurement, and glucose levels. If an infection is suspected, the analysis should include venereal disease reaction level, Gram stain, fungal and acid-fast bacilli tests, and culture with antibiogram. At the physician’s discretion, other analyses may be included (e.g., neoplastic cell search, specific immunological reactions, or oligoclonal band search).

## BLOOD-BASED BIOMARKERS

In recent years, there has been significant improvement in the technologies that enable the measurement of Aβ peptide and different epitopes of p-tau in plasma. The latest availability of plasma assays for these biomarkers was made possible by developing more precise techniques, as the plasma concentration of these proteins is much lower than in CSF. The use of assays based on mass spectrometry or more sensitive immunoassays (e.g., SIMOA™) has made it a reality to measure these biomarkers in plasma^42,68,69^.

Compared to well-established CSF and PET methods, plasma biomarkers offer a less invasive, more feasible, and potentially more cost-effective option in clinical practice. Additionally, they have the potential to significantly reduce screening time and costs in clinical trials and can be used as surrogate outcomes to evaluate the efficacy of therapies. However, the validation of these plasma biomarkers is still in development, and currently, they cannot be used alone for diagnosis. While a recent study suggests the possible superiority of plasma over CSF biomarkers^45^, another study using machine learning to predict AD-associated cognitive decline suggests the superiority of CSF biomarkers^70^.

Besides the mixed results regarding the superiority of plasma biomarkers for AD diagnosis, the limitation of real-world clinical validation, lack of studies in ethnically diverse populations and local norms (e.g., for the Brazilian population), and some methodological issues (pre-analytical care protocols and QC programs like those existing for CSF biomarkers) hinder the applicability of plasma biomarker^68^. For all these reasons, the systematic and routine use of plasma biomarkers in clinical practice is not currently recommended. Additionally, none of the plasma biomarker assays have been approved by the FDA as of June 2024^4^.

### Plasma Aβ_42_, Aβ_40_ and Aβ_42_/Aβ_40_ ratio

The development of various immunoprecipitation methods based on mass spectrometry has greatly improved the Aβ_42_/Aβ_40_ ratio accuracy. However, accuracy varies among the different methods used. While mass spectrometry-based assays have an area under the curve (AUC) ranging from 0.76–0.87, immunoassay methods have lower performance, with AUC ranging from 0.64–0.78^48^. This significant variability in accuracy between assay types is one of the main limitations to the clinical use of plasma Aβ peptide biomarkers^48,71^. The measurement of Aβ_42_ alone has not shown good accuracy and is therefore not performed; the Aβ_42_/Aβ_40_ ratio is always recommended.

An inherent problem with the plasma Aβ_42_/Aβ_40_ ratio, regardless of the method used, is that AD patients show only a small reduction in this parameter compared to controls. For reference, in the plasma of AD patients, the Aβ_42_/Aβ_40_ ratio is reduced by 8–15% compared to controls, while in CSF, the reduction is 40–60%^42,68,71^. This makes the plasma test less robust than the CSF Aβ_42_/Aβ_40_ ratio. One possible explanation for this difference is that the reduction in the Aβ_42_/Aβ_40_ ratio quantified in plasma reflects only a fraction of the Aβ detected in the blood, as the test does not distinguish between Aβ derived from the brain and that of extracerebral origin, the latter presumably not affected by AD pathology^42,68^. Developing plasma biomarkers that reflect the brain-derived fraction of Aβ remains a challenge.

Other important issues include the need for “real-world” studies to verify the robustness of the test, studies that validate it in diverse populations, and an understanding of intra-individual variability and disease-associated variability, as well as the potential impact of comorbidities and medications^42,68^. For these reasons, it is not recommended to use the Aβ_42_/Aβ_40_ ratio alone as the sole amyloid biomarker, for example, to indicate anti-amyloid therapy^42,68^. Plasma biomarkers are not recommended as unique diagnostic criteria for anti-amyloid therapy.

Combining the Aβ42/Aβ40 ratio with other biomarkers can improve its accuracy. West et al. designed a mass spectrometry platform to quantify plasma levels of Aβ_42_ and Aβ_40_ and identify specific peptides of the apolipoprotein E (ApoE) isoform — proteotyping, an indirect method of determining ApoE genotype^72^. In this study, the accuracy of Aβ_42_/Aβ_40_ increased from 81 to 86% when combined with ApoE proteotyping. The authors also developed an amyloid probability score (APS) by combining the Aβ_42_/Aβ_40_ ratio, ApoE proteotyping, and the patient’s age. The scores are categorized as low (0–35), intermediate (36–57), and high (58–100), corresponding to the likelihood of amyloid PET positivity, with a sensitivity of 84.9% and a specificity of 96.0%^73^. Although this platform is already being commercialized, it is emphasized that the measurement methods for Aβ_42_ and Aβ_40_ peptides still require better validation, particularly in the Brazilian population. Another aspect to consider in a blood matrix is identifying confounding factors that affect levels and their clinical utility before routine implementation. Recent evidence suggests that reduced renal function may be associated with increased plasma biomarker concentrations^74^. No platform for measuring Aβ_42_ and Aβ_40_ in plasma has received regulatory approval in the United States or the European Union^4^.

### Plasma phosphorylated tau

Similar to advancements in detecting plasma Aβ peptide through mass spectrometry, in recent years, several assays using this method have been developed for detecting p-tau in the blood. However, these assays are not yet validated and standardized for routine use (real-world studies) outside the context of clinical research. An inherent problem with p-tau is its extremely low plasma concentration. This consideration is necessary for interpreting study results and, obviously, for considering its application in clinical practice. Significant progress was achieved in p-tau measurement techniques, and in some cases, accuracy values equivalent to tau PET and even superior to CSF have been achieved^45^.

The referenced assays use antibodies to detect tau epitopes, as the intact protein is even less available in peripheral blood due to proteolytic processes. These epitopes are named according to the position of the amino acid at which they are phosphorylated. The most widely used epitopes in assays are p-tau181, p-tau217, and p-tau231. Tests with each of these variants have shown that they can differentiate patients with and without AD pathology^56,75,76,77^. Studies with neuropathological information have shown that plasma levels of p-tau217 reflect both the density of NFTs and amyloid plaques^78^. Notably, the increase in levels of these p-tau forms seems specific to AD and does not occur in other tauopathies such as corticobasal degeneration (CBD), progressive supranuclear palsy (PSP), or frontotemporal lobar degeneration (FTLD)^68,75^. Both p-tau181 and p-tau217 have also shown excellent accuracy in predicting the progression of patients with MCI to a stage of dementia due to AD^56,75^. On the other hand, p-tau231 is the first to change on the AD *continuum*, indicating a closer association with amyloid pathology than with tau pathology itself ^79^.

Of all the isoforms, p-tau217 has shown the highest sensitivity and specificity for the diagnosis of AD^80^. Unlike the Aβ_42_/Aβ_40_ ratio, which decreases by less than 15% in AD patients, p-tau217 increases by 300 to 700%^42^. This makes p-tau217 a much more robust test for detecting AD compared to the Aβ_42_/Aβ_40_ ratio. In a study comparing ten different plasma p-tau assays, the measurement of p-tau217 using a mass spectrometry-based assay presented an AUC of 0.947 and a correlation of 0.891 with CSF p-tau concentration^56^. Interestingly, p-tau217 levels also showed an excellent correlation with amyloid pathology^42^. Brum et al. developed an algorithm using p-tau217, ApoE genotyping, and age as a screening test to determine Aβ pathology positivity in patients with MCI^81^. This algorithm could predict amyloid pathology in 80% of cases (in the remaining 20%, the Aβ_42_/Aβ_40_ ratio in CSF was needed). In another study with 786 participants from three cohorts using an immunoassay, plasma p-tau217 concentration had high accuracy in predicting Aβ pathology (AUC=0.92–0.96; 95%CI 0.89–0.99) and tau pathology (AUC=0.93–0.97; 95%CI 0.84–0.99)^76^. Despite the need for more real-world validation studies, plasma p-tau217 (especially when using mass spectrometry-based assays) emerges as a promising biomarker for detecting proteinopathies (amyloid and tau)^45^. The ratio of plasma p-tau217 concentrations to phosphorylated p-tau at different sites (np-tau217) has been used in some studies to further increase the diagnostic accuracy of p-tau217^82,83^. Most recently, an algorithm combining the plasma Aβ_42_/Aβ_40_ ratio and the percentage of p-tau217 relative to np-tau217 to produce an amyloid probability score 2 (APS2) had accuracy of 88% (AUC=0.94; 95%CI 0.92–0.96), with 88% agreement with amyloid PET^84^.

As with plasma Aβ, the presence of certain comorbidities can significantly affect the plasma concentration of tau epitopes. Understandably, chronic kidney disease — once it affects protein clearance—is associated with higher levels of p-tau181 and p-tau217. Additionally, conditions such as hypertension, stroke, and myocardial infarction also elevate these levels^85^. These conditions may presumably modify the safe cutoff points for the test, but this relationship has not yet been established.

## NEUROIMAGING BIOMARKERS

Among the neuroimaging biomarkers for evaluating AD, structural neuroimaging methods such as computed tomography (CT) and magnetic resonance imaging (MRI) stand out, along with nuclear medicine techniques.

Molecular neuroimaging and nuclear medicine methods include conventional brain scintigraphy, acquired in gamma cameras in three-dimensional form (single-photon emission computed tomography — SPECT), and the PET. In these exams, the radioactive source emanates from the patient, who receives an intravenous injection of radiotracers. These procedures are safe, with no significant adverse events or reported allergies. PET has a higher spatial resolution than SPECT. These exams are currently performed on hybrid multimodal machines (SPECT/CT, PET/CT, and, more recently, PET/MRI) to compensate for their lack of spatial resolution. Today, almost all PET machines have a PET/CT configuration^86^.

These methods are highly sensitive for selecting patients for anti-amyloid therapies and for prognosis evaluation/stratification. They are commonly used for outcome assessments in most recent AD studies, mainly due to their ability to detect specific pathological or physiological changes. However, their use is limited in monitoring the safety of new anti-amyloid therapies compared to MRI, the gold standard in neuroradiology. MRI has recognized superiority in detecting general conditions such as bleeding, stroke, edema, and amyloid-related imaging abnormalities (ARIA)^87^.

MRI is very useful in the initial evaluation of individuals with dementia due to its ability to detect structural causes (e.g., cerebrovascular disease) or potentially reversible causes (e.g., benign neoplasms) of cognitive decline^88^. In some cases, especially when MRI is not accessible, CT can be used as a substitute. However, RM use is not limited to initial evaluation; it can also be employed as a biomarker of neurodegeneration, as highlighted hereinafter.

Imaging exams can function as biomarkers for AD throughout the entire AT(N) staging^44^. Using PET scans with amyloid or tau tracers, it is possible to detect the cerebral deposition of Aβ peptide plaques (“A” staging) or intracellular tangles of phosphorylated p-tau (“T” staging), respectively, as well as their removal after treatments. While fluid biomarkers (CSF and plasma) reflect the production or clearance of the soluble forms of proteinopathies, PET tracers mark the insoluble aggregates, measuring the cumulative effects of the proteinopathies and providing information on the neuroanatomical distribution of the pathology^89,90^.

PET scans assessing glucose metabolism [^18^F]-Fluorodeoxyglucose (FDG-PET), cerebral perfusion by SPECT, and MRI can identify signs of neurodegeneration (“N” staging)^91^. FDG-PET, besides being an N biomarker for investigating AD, can aid in differential diagnosis by demonstrating neurodegeneration patterns suggestive of AD or other non-AD degenerative diseases, as outlined by the NIA-AA Research Framework^43^ and confirmed in a Brazilian study^92^. Furthermore, FDG-PET is particularly useful for characterizing atypical forms of AD (logopenic variant, posterior cortical atrophy, and dysexecutive-behavior variant).

In Brazil, the main limitations of molecular neuroimaging assessment are the unavailability of certain tracers (especially tau PET), the high cost combined with the limited availability of others (amyloid PET) in routine clinical practice, and, most notably, the high cost of combining multiple tests, as detailed thereafter.

### Amyloid plaque marker positron emission tomography

The PET scan with an amyloid plaque marker (amyloid PET) represents a significant technical advancement in AD imaging assessment, being included as an “A” staging marker in the latest diagnostic criteria^4,44,49^.

This method detects extracellular cerebral deposits of Aβ peptide in moderate/frequent neuritic plaques with high sensitivity and specificity (96% and 100%, respectively)^2,93^. A normal amyloid PET scan excludes AD as a diagnosis in patients with cognitive impairment. Therefore, it is indicated as a prerequisite to confirm or exclude a clinical syndrome related to AD, with high specificity and reproducibility, making it an excellent tool for selecting patients for clinical trials and approved treatments.

There are four most common and commercially available amyloid PET tracers. One of them, labeled with carbon-11, ^11^C-Pittsburgh compound-B ([^11^C]-PiB), is considered open-access; however, it requires the presence of a cyclotron at the exam location due to the very short half-life of carbon-11. The other three tracers, labeled with fluorine-18, have commercial use due to the longer half-life of fluorine-18 and were approved by European and United States regulatory agencies for clinical use: [^18^F]-Florbetaben, [^18^F]-Florbetapir, and [^18^F]-Flutemetamol. Currently, the tracers available in Brazil are [^11^C]-PiB and [^18^F]-Florbetaben.

**Figure 1:**
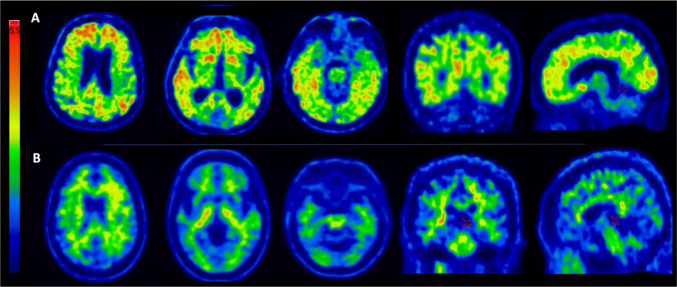
Axial images of amyloid PET of two individuals with [11C]-PiB showing significant cortical uptake of the tracer (A - upper row - a “positive” scan, or A+) and absence of significant uptake of the tracer (B - lower row - a “negative” scan, or A-). In the A+ individual, a diffuse uptake is seen in the frontal, parietal, and temporal neocortices, as well as in the medial parietal and frontal lobes (precuneus, posterior and anterior cingulate gyrus), with an intensity similar to or more intense than the physiological uptake in the white matter. A negative scan (B) shows an uptake restricted to white matter tracts. Red localizers show the correct way to visualize the medial parietal and medial frontal cortices in the medial views (at the cortex level as localized in the coronal or axial views). Note the absence of uptake in the cerebellar cortex (a reference for negativity) and the physiological uptake in the brain stem (a reference for positivity).

Source: Images from the personal archive of one of the authors (A.M.C.) and acquired at the Nuclear Medicine Center of the Institute of Radiology, Hospital das Clínicas da Faculdade de Medicina da Universidade de São Paulo (HCFMUSP), São Paulo, SP, Brazil.

Amyloid PET scans are generally dichotomous and classified as positive or negative (A+ or A-), facilitating the selection of individuals for clinical studies (Figure 1). Negative tests (A-) rule out the possibility of AD. The incidence of A+ in cognitively normal individuals can range from 2.7% in people aged 50 to 59 years to 41.3% in people aged 80 to 89 years^94^, typically varying around 10–20% in individuals aged between 60 and 70 years. Conversely, about 25–30% of individuals with a clinical diagnosis of AD have negative (A-) scans in most studies, indicating a high percentage of patients with clinically suggestive AD but with another underlying pathology^95^. There are minimal variations in the percentage of A+ between cohorts and concordant results with international studies using [^11^C]-PiB. The Brazilian setting demonstrates 18% positivity among controls (mean age ± standard deviation [SD] = 71.19±6.1 years) and 76% in individuals with clinically suggestive AD (mean age ± SD = 73.7±7.3 years)^92^, highlighting the limitation of solely clinical diagnosis of the disease.

In response to the initial results of studies with amyloid PET, joint recommendations from the Society of Nuclear Medicine and the AA for the use of amyloid PET were published in 2013^96^. This consensus recommended the method in the following cases:

- Persistent or progressive MCI of unexplained cause;
- Patients with clinical criteria for possible AD, but with atypical clinical presentation or possible mixed etiology; and
- Patients with progressive dementia and atypical age of onset (pre-senile), defined as symptom onset at 65 years or younger.

The same recommendations suggested not using amyloid PET in the following situations:

- Patients who meet clinical criteria for probable AD with typical age of onset;
- To determine the severity of dementia;
- Based solely on a family history of dementia or the presence of the ApoE ε*4* allele;
- Patients with SCD;
- As a substitute for genotyping in carriers of autosomal dominant mutations;
- Asymptomatic individuals; and
- Non-medical use (e.g., legal purposes, insurance coverage evaluation, employment, or occupational assessment).

It is important to note that these recommendations were published in 2013, before the adoption of biological diagnostic criteria for AD research based on biomarkers.

Amyloid PET scans have been included as biomarkers used as biological outcome variables in all clinical trials of anti-amyloid drugs approved so far on the United States market to monitor the biological success of anti-amyloid therapy. Based on their high specificity, the reduction of amyloid load was considered one of the aspects that contributed to the approval of these drugs^97,98,99,100,101^. In October 2023, the Centers for Medicare & Medicaid Services in the United States approved coverage for one amyloid PET scan during the lifetime of patients with AD, primarily to appropriately select patients for anti-amyloid treatment^102^. Regarding clinical use for early detection or differential diagnosis of neurodegenerative diseases, some authors have suggested the ideal timing for using this method, including its optimal timing relative to other molecular imaging methods^2,86^.

The interpretation of typical images obtained with amyloid PET depends on the knowledge of the progression of amyloid deposition and its topography during the course of AD. Initially, plaques form in the neocortex (frontal, parietal, temporal, occipital) (Thal phase 1); next, in the allocortex (entorhinal cortex, subiculum, hippocampus [CA1]), cingulate cortex, and amygdala (Thal phase 2); then the basal ganglia, thalamus, and hypothalamus are affected (Thal phase 3); and finally, the midbrain, pons, and cerebellum (Thal phases 4 and 5)^25,103,104^.

### Tau protein positron emission tomography

PET scans with p-tau deposit tracers are not yet part of routine clinical practice, even in centers in developed countries. However, research results indicate that this exam may eventually be included in clinical practice due to its potential to replace older exams by combining the detection of pathological processes with disease staging and differential diagnosis^86,105,106,107^.

In the revised criteria for diagnosis and staging of AD, published by AA workgroup, it is clearly stated that tau PET can discriminate between biological stages, categorized from A to D, whereas fluid biomarkers can only establish that an individual is in stage A or higher. Stage A corresponds to positive amyloid PET and negative tau PET (A+T_2_); tau PET with uptake only in the medial temporal region (A+T_2MTL_+) is equivalent to stage B; tau PET with moderate neocortical uptake (A+T_2MOD_+) is stage C; and high tau PET high neocortical uptake (A+T_2HIGH_+) represents stage D. So, for staging AD, tau PET is currently the only available biomarker^4^.

P-tau tracers have been validated by *post-mortem* pathological studies, showing a strong association with NFTs^47,89,107^. However, studies comparing p-tau levels in fluids with tau PET have shown that p-tau increases in both CSF and plasma before tau PET becomes positive^42^. Despite this, tau PET is highly associated with the neuroanatomical distribution of NFTs, thus reflecting the clinical manifestations, including the phenotypic variants of AD (i.e., non-amnestic presentations)^107^.

The interpretation of typical images obtained with tau PET depends on the topographical knowledge and progressive distribution or staging of NFT appearance in the course of AD. Initially, the transentorhinal stage is identified (Braak & Braak stages I–II), followed by the limbic stage (Braak & Braak stages III–IV), and finally the isocortical stage (Braak & Braak stages V–VI)^25,108^.

The first generation of tau tracers, most notably the radiopharmaceutical [^18^F]-FAV1451 (formerly [^18^F]-T807 and commercially known as [^18^F]-Flortaucipir), has affinity only for 3R/4R helical tau filaments, which are exclusive to AD. These methods are excellent for selecting T+ or T- individuals in AD and for staging the disease, as their deposition follows the Braak & Braak staging observed in pathological studies^109^, which can help assess disease progression. However, this marker has not been able to identify deposits of 3R or 4R tau related to primary tauopathies, which are mainly represented by FTLD, CBD, and PSP^110^. Currently, [^18^F]-Flortaucipir is the only p-tau tracer approved for clinical use in the United Sates^111^.

Second-generation tau tracers appear to overcome the limitations related to primary tauopathies and are mainly represented by [^18^F]-MK6240 and [^18^F]-PI2620. Their use allows for the identification of 3R/4R tau deposits in AD with high affinity, staging/stratifying AD, and differentiating it from other tauopathies both by the spatial distribution of tracer deposition and by lower affinity and intensity of uptake in 4R tauopathies^105,112,113^. This method could also evaluate the evolutionary stage as a staging marker in these conditions.

Once clinically tested and approved, these second-generation radiopharmaceuticals could be used to select individuals based on their pathological status (strongly T+ individuals are generally A+), perform differential diagnoses with other tauopathies, and simultaneously stratify and classify diseases with 3R/4R or 4R tau deposition. They could also help differentiate AD from both tauopathies and alpha-synucleinopathies, the latter presenting negative scans (absence of tracer deposition). Thus, this method has enormous potential for routine clinical use in the future. These tracers represent a significant advancement not only in selecting individuals for AD trials but also for anti-tau treatments in diseases such as PSP.

Tau PET methods are not currently available in Brazil, and there are no perspective for their clinical use in the short term. Additionally, the costs of these procedures are expected to be high. There are also no well-defined criteria for their visual analysis in clinical practice. This is a hindrance to the use of the newly proposed revised criteria for staging AD in clinical practice in Brazil and in many other countries.

### [^18^F]- Fluorodeoxyglucose positron emission tomography

**Figure 2:**
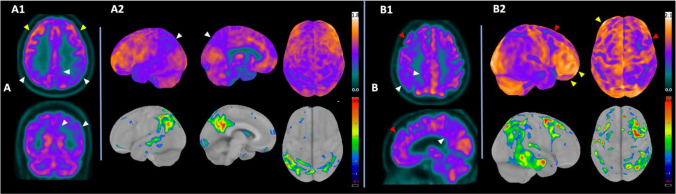
Typical pattern of neurodegeneration in Alzheimer’s Disease as seen on FDG-PET at different stages of progression in two individuals with predominantly amnestic presentation and confirmed cortical deposition of beta-amyloid plaques by [^11^C]-PiB PET. (A) Hypometabolism in the posterior temporal neocortex, temporoparietal associative cortex, posterior cingulate, and precuneus, slightly more evident on the left side, highlighted by white arrows. (A1) Images in axial and coronal planes, showing preserved frontal metabolism (yellow arrows). (A2) 3D-SSP* quantification images demonstrating in the top row the projection of metabolism on the cortical surface in left lateral, medial, and superior projections; in the bottom row, the projection of the Z-score of hypometabolic areas in relation to a normal control database by the same method is shown. (B) Patient in the moderate phase of the neurodegeneration process, with prefrontal hypometabolism (red arrows) in addition to hypometabolism in posterior temporoparietal regions (white arrows). Notable is the preservation of metabolism in polar and supraorbital frontal regions (yellow arrows), an important differentiation from other pathological processes (e.g., frontotemporal dementia). (B2) 3D-SSP* quantification images demonstrating in the top row the projection of metabolism on the cortical surface in right lateral and superior projections; in the bottom row, the projection of the Z-score of hypometabolic areas in relation to a normal control database is shown.

Source: Images from the personal archive of one of the authors (A.M.C.) and acquired at the Nuclear Medicine Center of Institute of Radiology, Hospital das Clínicas da Faculdade de Medicina da Universidade de São Paulo (HCFMUSP), São Paulo, SP, Brazil.

Note: *3D-SSP images obtained by Cortex ID Suite, GE Healthcare. Color scales to the right – upper bars in standardized uptake value ratio (SUVr) with the global cortex mean as the normalization reference; lower bars in Z-score values relative to an age-matched normal control database.

FDG-PET remains the primary molecular neuroimaging method available in clinical practice. The pattern of regional hypometabolism in the temporoparietal association areas, medial temporal, precuneus, and posterior cingulate shows high sensitivity and specificity in indirectly detecting AD-related pathology (Figure 2). The accuracy is around 90% in AD or slightly higher in various recent cohorts, including in the prediction of *in vivo* amyloid in national series, both in individuals with a broad spectrum of amnestic syndrome^92^ and in non-amnestic variants such as corticobasal syndrome^114^. It is important to note that the test shows higher accuracy in the dementia phase or in cases of MCI due to AD in more advanced stages of neurodegeneration, and lower accuracy in the early stages of MCI due to AD.

On the other hand, while a normal FDG-PET scan does not exclude the diagnosis of a degenerative disease, it suggests a favorable prognosis of potential cognitive stability over an average follow-up period of three years^115^. Additionally, the combination of PET with CT in PET/CT equipment provides the advantage of simultaneously evaluating cerebral metabolism, structural changes, and vascular load. Although this is less refined than MRI, it is often sufficient for clinical purposes.

Ossenkoppele et al. indicated that the neuroanatomical pattern of hypometabolism in FDG-PET showed a strong negative correlation with areas of tracer hyper-uptake for p-tau. In other words, the hypometabolism pattern (neurodegeneration) reflects the distribution of tau pathology in AD^106^. This overlap between p-tau accumulation (tau PET) and areas of hypometabolism (FDG-PET) has a significant implication: FDG-PET can be a good substitute for tau PET in locations where it is unavailable^43,92^. However, it is important to note that, according to the amyloid cascade hypothesis, tau PET changes precede those observed in FDG-PET, a marker of neurodegeneration. In *post-mortem* analyses in which FDG-PET was evaluated by physicians who were not molecular imaging specialists, the exam showed a sensitivity of 80% and a specificity of 84%^40^. Previous studies, however, demonstrated wide variability in these values, with sensitivity ranging from 88–98% and specificity from 80–95%^40^. Generally, older studies, in which exams were not reviewed by specialists or did not use quantitative methods in clinical practice, presented more modest results.

Motara et al. evaluated the clinical impact of using FDG-PET according to its indications: diagnostic difficulty after formal clinical procedures and inconclusive structural analysis, pre-senile dementia, differentiation between AD and FTD, atypical cases of AD and FTD, psychiatric comorbidities associated with cognitive decline, and inconclusive neuropsychological evaluation^116^. In this evaluation, FDG-PET impacted clinical management in 81% of individuals (79/98), changing the pre-test diagnosis in 35% of cases, reducing the need for further investigations by 42%, and altering therapy in 32%. These results are similar to those of Laforce Jr et al., who showed a clinical management impact of 56%, with a diagnosis change in 29% of cases^117^.

A consensus article based on expert opinions from the European Associations of Nuclear Medicine (four individuals) and Neurology (three individuals) determined in 2018 several clinical situations in the context of neurodegenerative diseases where FDG-PET had sufficient evidence to recommend its clinical use, in addition to clinical/neuropsychological examination. Naturally, there were differences in the degree of evidence and recommendation of indication among the evaluators, but ultimately, FDG-PET was recommended in several circunstances:

- To aid in the diagnosis of atypical AD;
- To differentiate AD from DLB, FTD, and vascular dementia;
- To differentiate DLB from FTD, and Parkinson’s disease from PSP; and
- To suggest pathophysiology in corticobasal syndrome, primary progressive aphasia, and assess cortical dysfunction in Parkinson’s disease.

In all indications, the routine use of semi-automated quantification to assist visual analysis was recommended, which has been internalized in the European guidelines for performing the exam^118^. The indications of FDG-PET related to AD were those where in general, there was the greatest consensus among specialists^119^.

Cerebral perfusion assessment by SPECT can be a more cost-effective alternative to FDG-PET but has several disadvantages, including lower spatial resolution and accuracy^120^, fewer commercially available semiquantitative programs for the method, and the lack of integrated CT in most available machines. However, SPECT can be useful for differential diagnosis between AD and FTD, provided that the exam is interpreted by an experienced nuclear medicine physician^121^. Additionally, the accuracy of SPECT is not affected by glycemic control or diabetes, which can potentially influence FDG-PET results^122,123^.

### Magnetic resonance imaging as a marker of neurodegeneration

In addition to its role in excluding reversible and irreversible structural causes of cognitive decline, MRI can also be used as a biomarker of neurodegeneration. However, its accuracy in this evaluation is generally inferior to FDG-PET when only visual analysis of structural images is used. Structural changes occur later than those detected by other biomarkers, except for cognitive markers, which may appear even later or coincide with the structural changes observed on MRI.

However, MRI shows a significant increase in accuracy when its analysis is optimized using quantitative analysis programs, such as those for measuring gray matter volume or cortical thickness reduction. Initially available in research settings, these programs have recently been adapted for commercial use. More specifically, the most commonly used neurodegeneration marker of atrophy of the medial temporal lobe is preferably characterized by the medial temporal lobe atrophy (MTA) score^124^. The interpretation of the MTA score is based on age and degree of atrophy, with a score above 1 considered abnormal for patients up to 74 years and a score above 2 considered abnormal for patients aged 75 years or older. Other scales used for measuring the atrophy commonly found in AD patients are the Koedam and entorhinal cortical atrophy scale (ERICA), respectively quantifying parietal and entorhinal regions.

## BIOMARKERS IN ETHNICALLY DIVERSE POPULATIONS

Despite the recent increased availability of biomarkers, many of the studies validating cutoff points have been conducted in populations of white people living in high-income countries, like the United States and European countries. Biomarkers seem to behave differently in black individuals^42,125,126^. Recent clinical trials of anti-amyloid therapies that used AD biomarkers as inclusion criteria had a large disproportion of black participants who did not meet the inclusion criteria based on these biomarkers, suggesting that the cutoff points in this population may differ^99,101^. In the lecanemab clinical trial, only 2% of the participants were black^99^.

## PERSPECTIVES

The field of biomarker research has grown significantly. Certainly, the development of plasma biomarkers will make AD diagnosis more accessible and less expensive. However, the quality of studies remains variable. Recently, the Global CEO Initiative on Alzheimer’s Disease convened a BBM Workgroup to consider the minimum acceptable performance of blood-based biomarkers (BBM) tests for clinical use^127^. A BBM test should have a sensitivity ≥90% with a specificity ≥85% in primary care and ≥75–85% in secondary care in case of screening test prior to subsequent confirmatory testing (PET or CSF). If it is used as a confirmatory test, a plasma biomarker should have performance equivalent to that of CSF tests (with sensitivity and specificity of at least ~90%)^127^.

The BBM Workgroup also suggests that plasma biomarkers could have two cutoffs to define three categories of result: positive, intermediate, and negative^127^. Patients with a result below the lower cutoff (negative) are highly likely not to have AD, and those with a result above the upper cutoff (positive) are highly likely to be diagnosed with AD. In cases where the result is between the two cutoffs (intermediate), a confirmatory test (PET or CSF) or a repeat plasma biomarker should be considered in one year. However, the BBM Workgroup does not yet endorse any specific test^127^.

In addition to the improvement of Aβ and p-tau pathology biomarkers, there is a search for biomarkers related to other aspects of pathology. For example, biomarkers of neuroinflammation associated with AD, such as markers of astrocytic and microglial activation. Among these, the plasma measurement of glial fibrillary acidic protein (GFAP), expressed in astrocytes, stands out^42,128,129^. Similar to fluid-based biomarkers of neuroinflammation, molecular neuroimaging for neuroinflammation has shown promise with several studies in the last decades^130,131,132,133,134,135^. Radiopharmaceuticals have been developed, some that demonstrate both microglial activation (e.g., [^11^C]-PK11195)^131,133^, and others that show astrocytic activity (e.g., [^11^C]-Acetate)^134,135^. However, PET for neuroinflammation is still restricted to research, with no applicability in clinical practice to date.

Another area of intense study is the search for biomarkers of other proteinopathies and cerebrovascular diseases that frequently occur as co-pathologies in patients with AD. Among these, alpha-synuclein is one of the most relevant^136^.

## PRACTICAL RECOMMENDATIONS FOR REQUESTING BIOMARKERS IN CLINICAL PRACTICE

Biomarkers for AD are not yet perfect but play an important role in aiding the clinical diagnosis in symptomatic patients, particularly those with atypical presentations, and have implications for indicating disease-modifying therapy. The request and interpretation of biomarkers are not trivial and should be performed by physicians well-trained in the field, usually from the medical specialties of Geriatrics, Neurology, Psychiatry (Old-Age Psychiatry). Biomarkers should only be requested for cognitively symptomatic patients with objectively demonstrated deficits in cognitive testing, i.e., with a clinical dementia rating (CDR) score of 0.5 or above. They may be also requested in FTD patients, with significant behavioral changes for differential diagnosis. Biomarkers can be indicated when it is necessary to make a precise etiological diagnosis of cognitive impairment.

Thus, biomarkers for AD are not indicated in asymptomatic individuals, patients with SCD, or in cases of advanced dementia (where etiological diagnosis will not change clinical management).

Biomarkers should not be requested to predict the risk of developing cognitive decline in asymptomatic individuals for several reasons:

- There is no disease-modifying treatment for the preclinical phase; and
- Not all individuals with positive biomarkers will progress to MCI or dementia stages in AD.

However, biomarkers can be recommended in the following clinical contexts:

- Early-onset dementias (symptoms onset before 65 years of age);
- Rapidly progressive dementias;
- Atypical dementia;
- Available disease-modifying treatment (e.g. anti-amyloid therapy); and
- Differential diagnosis with other neurodegenerative dementias^41,137^.

Biomarkers can also be recommended when patients want to know if their cognitive decline is caused by AD^138^. In all situations mentioned above, the interpretation of biomarkers should be done with caution, in the context of the clinical picture, and by a clinician with solid experience in the area.

Therefore, requesting biomarkers for AD is not a trivial procedure and should not be performed in the absence of clinical symptoms. It should also not be done by medical professionals who are not trained to correctly interpret the results and manage the consequences of the diagnosis for the patient and family.

The current objective of biomarkers for AD is to assist in the diagnosis of symptomatic patients. A future objective may be the monitoring of therapeutic response and disease progression. The diagnosis of AD based on biomarkers has important implications for treatment with new disease-modifying therapies, being fundamental in the indication or contraindication of these therapies. Table 3 summarizes the commercially accessible biomarkers for clinical use (some of which are not yet available in Brazil). We recommend that laboratories provide the ratios between analytes in CSF (Aβ_42_/Aβ_40_, p-tau181/Aβ_42_) as they have better accuracy than the isolated value of the analyte (especially in the case of Aβ_42_).

**Table 3:** Currently available biomarkers for clinical use.

Category	CSF biomarkers	Plasma biomarkers	Neuroimaging biomarkers
Proteinopathy Aβ (A)	↓ Aβ_42_/Aβ_40_ ↑ p-tau/Aβ_42_	↓ Aβ_42_/Aβ_40_	Positive amyloid PET [^11^C]-PiB, [^18^F]-Florbetaben, [^18^F]-Florbetapir*, [^18^F]-Flutemetamol*
Proteinopathy phosphorylated tau (T)	↑ p-tau181 ↑ p-tau181/Aβ_42_	↑ p-tau 217* ↑ p-tau217/np-tau217	Positive tau PET ([^18^F]-Flortaucipir*)
Neurodegeneration (N)	↑ NfL ↑ t-tau	↑ NfL* ↑ t-tau	FDG-PET with temporoparietal, posterior cingulate and precuneus hypometabolism Brain MRI with medial temporal lobe atrophy (particularly the entorhinal cortex), precuneus and temporoparietal cortex.

Abbreviations: CSF, cerebrospinal fluid; Aβ, amyloid beta; p-tau: phosphorylated tau; PET, positron emission tomography; NfL, light chain neurofilaments; FDG, [^18^F]-Fluorodeoxyglucose; MRI, magnetic resonance imaging.

Note: *Biomarkers not commercially available in Brazil.

The validation of some biomarkers, especially plasma analytes, is in the process of development, particularly in populations underrepresented in clinical research, which is generally conducted in high-income countries with a predominance of white participants.

**Figure 3:**
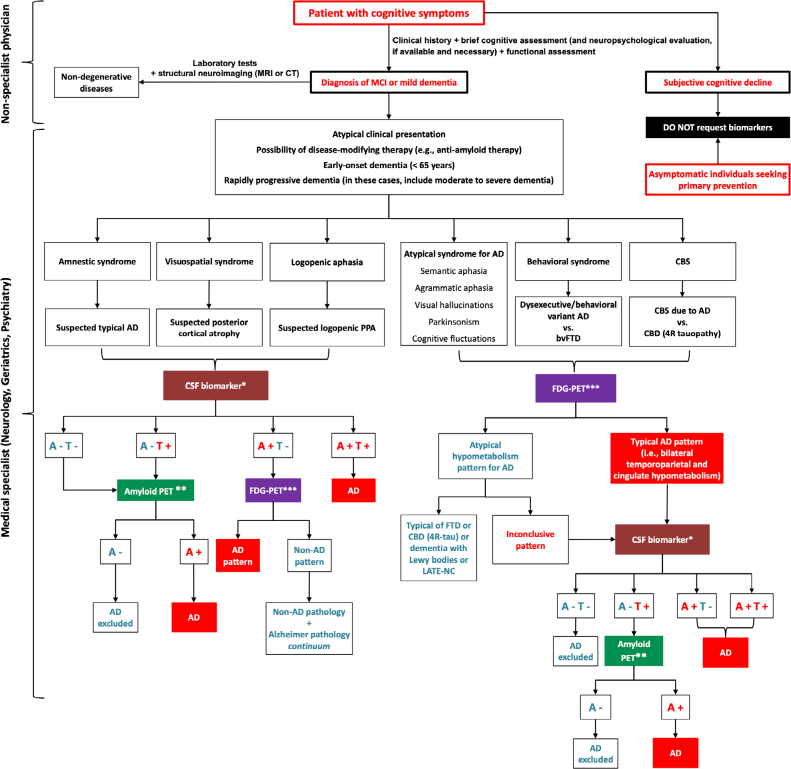
Suggestion for a rational use algorithm of biomarkers based on typical variant, and atypical cognitive syndromes of Alzheimer’s disease. At present, plasma biomarkers require better validation and, therefore, have not been included in this flowchart.

Abbreviations: AD, Alzheimer’s disease; MCI, mild cognitive impairment; PPA, primary progressive aphasias; MRI, magnetic resonance imaging; CT, computed tomography; PET, positron emission tomography; FDG, fluorodeoxyglucose; FTD, frontotemporal dementia; CBD, corticobasal degeneration; LATE, Limbic-predominant age-related TDP-43 encephalopathy.

Notes: *CSF biomarker should be performed on automated platforms (e.g., electrochemiluminescence immunoassay or mass spectrometry); **Caution should be exercised when interpreting amyloid PET in patients aged >80 years, as they may be positive in cognitively normal individuals in this age group; ***In the absence of FDG-PET, a SPECT with quantification can be considered.

We have elaborated a flowchart to assist physicians in requesting biomarkers, as shown in Figure 3. Plasma biomarkers were not included in this algorithm because they still need to be validated and have a quality control certificate. These tests are promising, such as p-tau217, and once they are adequately validated, the algorithm will certainly be updated to include these plasma biomarkers. For a plasma biomarker to be indicated, it must demonstrate an accuracy of at least ~90% compared to an amyloid PET or an already approved CSF biomarker platform by regulatory agencies.

Regarding CSF biomarkers, we suggest verifying if the laboratory is compliant with pre-analytical and analytical procedures and if there is quality control as required by the Alzheimer’s Association. We also recommend that laboratories adopt automated platforms, such as electrochemiluminescence immunoassay, instead of the ELISA technique.

Regarding PET scans, we propose that they be conducted in clinics with experienced nuclear medicine physicians qualified at evaluating brain PET scans, and whose analysis includes semiquantitative methods, not just visual. We recommend avoiding amyloid PET and other amyloid research tests isolated in patients aged 80 or older due to the high positivity rate in asymptomatic individuals in this age group, except when the goal is to rule out AD diagnosis (i.e., negative result). We also consider that measuring biomarkers in CSF is more available and less costly than amyloid PET (though more invasive due to lumbar puncture), and therefore recommend CSF examination as the first option over amyloid PET. Finally, the request for biomarkers should be made after a structured clinical evaluation, including detailed history, physical examination, and cognitive and functional assessments, in addition to neuropsychological evaluation if necessary^137,139^.

Laboratory tests and structural neuroimaging (ideally cranial MRI, with CT as a second option) should always be requested for all patients to exclude non-degenerative etiologies. Much has been discussed about diagnosing AD exclusively through biomarkers (biological diagnosis), regardless of clinical manifestations^44,49^. However, we recommend that the search for an AD diagnosis should begin with the presence of symptoms and that the characterization of the cognitive-behavioral syndrome should be the starting point for selecting which biomarkers to request^7^. It should also be noted that in many cases of patients diagnosed with AD *in vivo*, *post-mortem* evaluation reveals multiple pathologies (e.g., alpha-synuclein, TDP-43, and cerebrovascular disease)^14,140,141^. This means that the positivity of an AD biomarker does not necessarily indicate that the patient’s symptoms are solely due to AD. Therefore, the interpretation of biomarkers should be made in the context of the clinical syndrome to avoid the risk of false diagnoses.

Finally, we recommend that validated CSF and PET biomarkers for clinical use be submitted to the Brazilian Health Regulatory Agency (ANVISA, *Agência Nacional de Vigilância Sanitária*) for regulatory approval in Brazil. This would enable their implementation in both the Brazilian Unified Health System (SUS, *Sistema Único de Saúde*) and the supplementary health system. Box 1 provides a summary of the main recommendations.

**Box 1. Recommendations from the Scientific Department of Cognitive Neurology and Aging of the Brazilian Academy of Neurology for the use and interpretation of Alzheimer’s disease biomarkers in clinical practice in Brazil.**

- Before requesting AD biomarkers, a structured clinical evaluation should be performed (detailed history, physical and neurological examination, and cognitive/functional assessment).
- Requests should only be made by well-trained physicians with solid experience in the field, usually from the following medical specialties: Geriatrics, Neurology, and Psychiatry (Old-Age Psychiatry).
- Requests should not be made by medical professionals who are not qualified to interpret the results and handle the consequences of the diagnosis for the patient and their family.
- Biomarkers should only be requested for cognitively and/or behaviorally symptomatic patients (objectively demonstrated in cognitive testing and clinical evaluation).
- Biomarkers for AD ***are not indicated*** in asymptomatic individuals and patients with subjective cognitive decline (cognitive complaints without test abnormalities)
- Biomarkers for AD ***are not indicated*** in cases of advanced dementia (except when the goal is to rule out AD diagnosis, which may change treatment; e.g., discontinuation of cholinesterase inhibitors or memantine).
- Biomarkers can be recommended in the following clinical contexts: early-onset dementias (symptoms onset before 65 years), rapidly progressive dementias, atypical AD manifestations (e.g., non-amnestic presentations), and other atypical clinical manifestations (e.g., when there is a suspected overlap of symptoms of two or more neurodegenerative dementias).
- Biomarkers can be recommended when there is suspicion of mild cognitive impairment or mild dementia due to AD and there is a possibility of indicating anti-amyloid therapy (or other disease-modifying therapies that may be approved in the future).
- Laboratory tests and structural neuroimaging (ideally cranial MRI, with CT as a second option) should always be requested for all patients to exclude non-degenerative etiologies.
- CSF biomarkers should be conducted in laboratories with the required pre-analytical and analytical procedures and quality control as required by the Alzheimer’s Association.
- Laboratories should adopt automated platforms (e.g., electrochemiluminescence immunoassay or mass spectrometry) instead of the ELISA technique.
- Laboratories should provide ratios between analytes in CSF (Aβ_42_/Aβ_40_, p-tau181/Aβ_42_) as they have better accuracy than the isolated value of the analyte (especially in the case of Aβ_42_).
- Plasma biomarkers still need to be validated and have a quality control certificate (an accuracy of at least close to 90% is recommended compared to an amyloid PET or a CSF biomarker platform already approved by regulatory agencies).
- We recommend caution in performing amyloid PET and other amyloid research tests in patients aged 80 or older due to the high positivity rate in asymptomatic individuals in this age group.
- We recommend CSF examination as the first option over amyloid PET (see flowchart in Figure 3), as measuring biomarkers in CSF is more available and less costly than amyloid PET.
- Regarding PET scans, we suggest to be performed in clinics with experienced nuclear medicine physicians who are experienced in evaluating brain PET scans, and whose analysis includes semiquantitative methods, not just visual.
- In the absence of FDG-PET, a SPECT with quantification can be considered.
- We recommend that validated CSF and PET biomarkers for clinical use be submitted to ANVISA for regulatory approval in Brazil.
- We recommend that ANVISA-approved biomarkers be implemented in both the Brazilian Unified Health System (SUS) and the supplementary health system.

## SUPPLEMENTARY MATERIAL

Diretrizes para o uso e interpretação dos biomarcadores de Doença de Alzheimer no Brasil: Recomen­dações do Departamento Científico de Neurologia Cognitiva e do Envelhecimento da Academia Brasileira de Neurologia, available at: https://www.demneuropsy.com.br/wp-content/uploads/2024/10/DN_2024C01_PT_15-10-aprovado.pdf.
